# Anti-malarial effect of gum arabic

**DOI:** 10.1186/1475-2875-10-139

**Published:** 2011-05-20

**Authors:** Adil Ballal, Diwakar Bobbala, Syed M Qadri, Michael Föller, Daniela Kempe, Omaima Nasir, Amal Saeed, Florian Lang

**Affiliations:** 1 Department of Physiology, University of Tübingen, Gmelinstr. 5, D-72076 Tübingen, Germany; 2 Department of Physiology, University of Khartoum, Elqasr Street, P.O.Box 102 Khartoum, Sudan

## Abstract

**Background:**

Gum Arabic (GA), a nonabsorbable nutrient from the exudate of *Acacia senegal*, exerts a powerful immunomodulatory effect on dendritic cells, antigen-presenting cells involved in the initiation of both innate and adaptive immunity. On the other hand GA degradation delivers short chain fatty acids, which in turn have been shown to foster the expression of foetal haemoglobin in erythrocytes. Increased levels of erythrocyte foetal haemoglobin are known to impede the intraerythrocytic growth of *Plasmodium *and thus confer some protection against malaria. The present study tested whether gum arabic may influence the clinical course of malaria.

**Methods:**

Human erythrocytes were *in vitro *infected with *Plasmodium falciparum *in the absence and presence of butyrate and mice were *in vivo *infected with *Plasmodium berghei *ANKA by injecting parasitized murine erythrocytes (1 × 10^6^) intraperitoneally. Half of the mice received gum arabic (10% in drinking water starting 10 days before the day of infection).

**Results:**

According to the *in vitro *experiments butyrate significantly blunted parasitaemia only at concentrations much higher (3 mM) than those encountered *in vivo *following GA ingestion (<1 μM). According to the *in vivo *experiments the administration of gum arabic slightly but significantly decreased the parasitaemia and significantly extended the life span of infected mice.

**Discussion:**

GA moderately influences the parasitaemia and survival of *Plasmodium-*infected mice. The underlying mechanism remained, however, elusive.

**Conclusions:**

Gum arabic favourably influences the course of murine malaria.

## Background

Gum Arabic (GA) from gummy exudates of *Acacia Senegal *[1] is a water-soluble [2] polysaccharide based on branched chains of (1-3) linked β-D-galactopyranosyl units containing α-L-arabinofuranosyl, α-L-rhamnopyranosyl, β-D-glucuronopyranosyl and 4-O-methyl-β-D-glucuronopyranosyl units [3]. It is considered one of the safest dietary fibers [4]. In Middle Eastern countries GA is employed in the treatment of patients with chronic renal disease and end stage renal failure [5]. Gum arabic increases the faecal nitrogen excretion [6] and decreases the production of free oxygen radicals [5].

Recent *in vitro *experiments revealed a powerful immunomodulary effect of GA on dendritic cells [7] antigen-presenting cells orchestrating the initiation of both innate and adaptive immunity and thus playing a pivotal role in the regulation of the immune response [8-11].

The intestinal fermentation of gum arabic leads to the formation of several degradation products including short-chain fatty acids [6]. Accordingly, GA treatment may enhance the serum butyrate concentrations [12]. Butyrate compounds have been shown to up-regulate the formation of foetal haemoglobin [13-15], which may in turn confer some protection against a severe course of malaria [16-18]. Specifically, foetal haemoglobin has been shown to delay the haemoglobin degradation and thus to impede the intraerythrocyte growth of *Plasmodium*. Accordingly, expression of foetal haemoglobin protects against a severe course of malaria [17,18].

Moreover, foetal haemoglobin may increase the susceptibility of foetal erythrocytes to oxidative stress [19]. As *Plasmodium falciparum *imposes oxidative stress on infected cells, it may trigger eryptosis, the suicidal death of erythrocytes [20,21]. Eryptosis is characterized by cell membrane scrambling with phosphatidylserine exposure at the cell surface [22-26]. The cell membrane scrambling is triggered by increased cytosolic Ca^2+ ^activity [23-27] and/or ceramide [28]. Ca^2+ ^enters erythrocytes through Ca^2+^-permeable cation channels, which are activated by osmotic shock, oxidative stress or energy depletion [29-33]. In addition, Ca^2+ ^stimulates Ca^2+^-sensitive K^+ ^channels [27,34,35], followed by cellular loss of KCl and osmotically obliged water leading to cell shrinkage [27]. The Ca^2+^-permeable cation channels are activated by oxidative stress [36], which thus stimulates eryptosis [37]. Excessive cytosolic Ca^2+ ^concentrations stimulate similarly apoptosis of nucleated cells [38].

Phosphatidylserine-exposing cells are recognized [39,40] and phagocytosed [41,42] by macrophages. Eryptotic cells are thus rapidly cleared from circulating blood [43]. The accelerated clearance of infected erythrocytes [44] may counteract the development of parasitaemia [45]. Enhanced susceptibility to eryptosis and accelerated clearance of *Plasmodium*-infected erythrocytes may confer relative protection against a severe course of malaria in carriers of sickle-cell trait, beta-thalassaemia-trait, homozygous Hb-C and G6PD-deficiency [46-50], in iron deficiency [21], as well as during treatment with lead [20], chlorpromazine [51] and cyclosporine [52]. The erythrocyte cation channel is inhibited by erythropoietin [53], which may again influence the course of malaria [54].

The present study explored, whether gum arabic favourably influences parasitaemia and host survival during malaria.

## Methods

Animal experiments were performed according to the German animal protection law and approved by the local authorities (registration number PY 2/06). Experiments were performed in healthy SV129/J wild type mice (aged 4 months, both male and female). The animals had free access to standard chow (ssniff, Soest, Germany) and drinking water. Murine erythrocytes were drawn from the animals by incision of the tail vein.

Human erythrocytes were drawn from healthy volunteers. The study was approved by the Ethical commission of the University of Tübingen.

*In vitro *experiments were performed at 37°C in Ringer solution containing (in mM) 125 NaCl, 5 KCl, 1 MgSO_4_, 32 N-2-hydroxyethylpiperazine-N-2-ethanesulfonic acid (HEPES)/NaOH (pH 7.4), 5 glucose, 1 CaCl_2 _[55]. Butyrate was added to the NaCl Ringer at final concentrations varying from 0.3 to 10 mM (Sigma, Schnelldorf, Germany). For *in vitro *treatment, the final haematocrit was adjusted to 0.3%.

For determination of phosphatidylserine exposure, FACS analysis was performed as described [29]. After incubation in the presence or absence of gum arabic, suspensions of *P. falciparum-*infected erythrocytes were stained with annexin V-APC (BD Biosciences Pharmingen, Heidelberg, Germany) and/or with the DNA/RNA specific dye Syto16 (Molecular Probes, Göttingen, Germany) to identify phosphatidylserine-exposing and infected erythrocytes, respectively. For annexin V-binding, erythrocytes were washed, resuspended in annexin V-binding buffer (Ringer solution containing 5 mM CaCl_2_. pH 7.4), stained with annexin V-APC (dilution 1:20), incubated for 20 min at room temperature, and diluted 1:5 with annexin V-binding buffer. Syto16 (final concentration of 20 nM) was added directly to the diluted erythrocyte suspension or co-incubated in the annexin V-binding buffer. Cells were analyzed by flow cytometry (FACS-Calibur, Becton Dickinson, Heidelberg, Germany) in fluorescence channel FL-1 for Syto16 (detected at 530 nm) and in FL-4 for annexin V-APC fluorescence intensity (detected at 660 nm).

For infection of human erythrocytes the human pathogen *P. falciparum *strain BinH [56] was grown *in vitro *[57]. Parasites were cultured as described earlier [58,59] at a haematocrit of 2% and a parasitaemia of 2-10% in RPMI 1640 medium supplemented with Albumax II (0.5%; Gibco, Karlsruhe, Germany) in an atmosphere of 90% N_2_, 5% CO_2_, 5% O_2 _[60,61].

For infection of mice *Plasmodium berghei *ANKA-parasitized murine erythrocytes (1 × 10^6^) were injected intraperitoneally [62,63] into wildtype mice. Where indicated gum arabic (10% in drinking water) was administered starting 10 days before the day of infection. Blood was collected from the mice 8 days after infection by incision of the tail. Parasitaemia was determined by Syto-16 staining in FACS analysis.

To estimate the *in vitro *growth of *P. falciparum*, the BinH strain was cultured and synchronized to the ring stage by sorbitol treatment as described previously [36]. For the *in vitro *growth assay, synchronized parasitized erythrocytes were aliquoted in 96-well plates (200 μl aliquots, 1% haematocrit, 0.5-2% parasitaemia) and grown for 48 h in the presence or absence of butyrate (0.3 mM - 10 mM). The parasitaemia was assessed at time 0 and after 48 h of culture by flow cytometry. Parasitaemia was defined by the percentage of erythrocytes stained with the DNA/RNA specific fluorescence dye Syto16.

To estimate DNA/RNA amplification of the intraerythrocytic parasite, the culture was ring stage-synchronized, and re-synchronized after 6 h of culture (to narrow the developmental parasite stage), aliquoted (200 μl aliquots, 2% haematocrit and 10% parasitaemia) and cultured for further 16 h in the presence or absence of butyrate (0.3 mM - 10 mM). Thereafter, the DNA/RNA amount of the parasitized erythrocytes was determined by Syto16 fluorescence as a measure of intraerythrocytic parasite copies.

Data are expressed as arithmetic means ± SEM and statistical analysis was made by t-test or ANOVA using Tukey's test as post hoc test, as appropriate. p < 0.05 was considered as statistically significant. The mouse survival was analysed utilizing the Kaplan-Meier estimator method.

## Results

According to a blood count, treatment with GA (10% in drinking water) did not significantly affect blood parameters in non-infected mice (Table 1).

**Table 1:** Arithmetic means (± SEM, n = 7) of erythrocyte parameters of noninfected and infected mice without or with gum arabic treatment (10% in drinking water)

	*Noninfected*		*Infected*	
	***- GA***	***+ GA***	***- GA***	***+ GA***
Erythrocyte number (10^6^/mm^3^)	10.78 ± 0.19	11.03 ± 0.10	6.79 ± 1.01^#, *^	8.32 ± 0.95^#, *^
Haemoglobin (g/dl)	15.57 ± 0.32	16.35 ± 0.16	9.47 ± 1.38^#, *^	11.77 ± 1.39^#, *^
Haematocrit (%)	43.34 ± 0.32	44.5 ± 0.39	28.27 ± 4.3^#, *^	34.31 ± 3.72^#, *^
Mean erythrocyte volume (MCV) (fl)	40.2 ± 0.38	40.47 ± 0.16	41.35 ± 0.63	41.37 ± 0.39
Erythrocyte haemoglobin concentration (MCHC) (g/dl)	35.91 ± 0.20	36.75 ± 0.13	33.88 ± 0.55^#^	34.04 ± 0.49^#^
^§^Haemoglobin/erythrocyte (pg)	14.4 ± 0.20	14.88 ± 0.07	14.01 ± 0.13	14.07 ± 0.10

^§ ^calculated from MCV and MCHC

* different from -GA

^# ^different from noninfected

**Figure 1:**
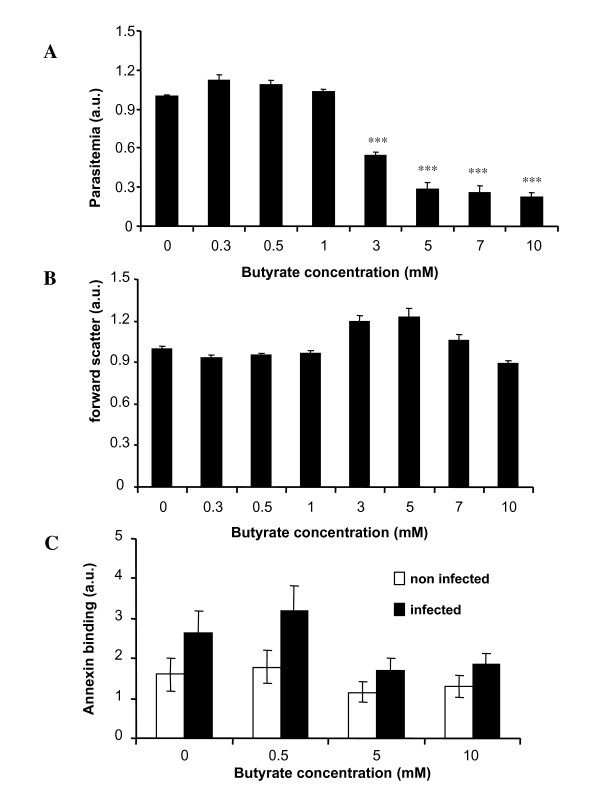
**Effects of butyrate during *in vitro *infection of human erythrocytes with *P. falciparum***. **A**. *In vitro *parasitaemia with *P. falciparum *in human erythrocytes as a function of the butyrate concentration (arithmetic means ± SEM, n = 3 cultures studied in quadruplets). **B**. Arithmetic means ± SEM (n = 3 cultures studied in quadruplets) of intraerythrocytic DNA amplification as a function of the butyrate concentration **C**. Arithmetic means ± SEM (n = 3 cultures studied in quadruplets) of the percentage of annexin V-binding infected (open bars) and non-infected (closed bars) erythrocytes following infection of human erythrocytes with *P. falciparum *in the presence of the indicated concentration of butyrate. *** (p < 0.001) indicate significant difference from absence of butyrate.

As treatment with GA is known to enhance plasma butyrate concentration, the influence of butyrate on the *in vitro *growth of the parasite was analysed. *Plasmodium falciparum*-infected erythrocytes were cultured in human erythrocytes and synchronized to ring stage by sorbitol treatment. Within 48 hours the percentage of infected erythrocytes increased from 5.09% to 17.82%. In the presence of butyrate, the increase in the percentage of parasitized erythrocytes was decreased, an effect reaching statistical significance at ≥ 3 mM butyrate concentration (Figure 1A). In contrast, the presence of butyrate did not show any significant decrease of the intraerythrocytic DNA amplification of the parasite (Figure 1B).

To determine the effect of infection and butyrate on suicidal erythrocyte death (eryptosis), the percentage of phosphatidylserine-exposing erythrocytes was estimated by measurement of annexin V-binding in FACS analysis. *In vitro *infection tended to increase the percentage of annexin V-binding erythrocytes (Figure 1C). The addition of butyrate tended to decrease the percentage of annexin V-binding cells, an effect, however, not reaching statistical significance (Figure 1C).

**Figure 2:**
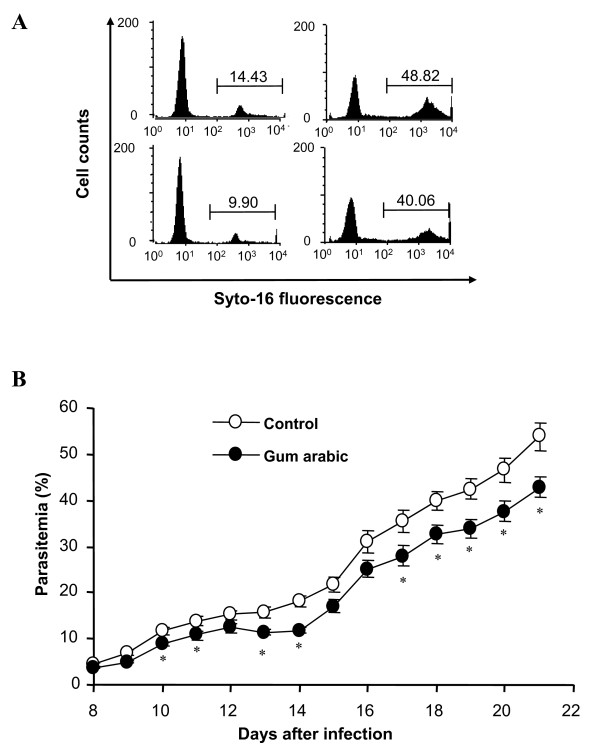
**Effect of gum arabic treatment on parasitaemia of *Plasmodium berghei*-infected mice**. **A**: Original histograms of parasitaemia-dependent Syto 16 fluorescence in untreated animals (upper panels) and animals treated from 10 days before infection until the day they survived with 10% gum arabic in drinking water (lower panels) 10 (left panels) and 20 (right panels) days after infection with *P. berghei*. **B**: Arithmetic means ± SEM of parasitaemia in mice without treatment (open circles, n = 19) or with 10% gum arabic in drinking water (closed circles, n = 17) as a function of days after infection with *P. berghei*. * (p ≤ 0.05) indicates significant difference from untreated animals.

To determine the *in vivo *efficacy of gum arabic, mice were infected with *P. berghei *with or without GA treatment. Gum arabic (10% in drinking water) was administered starting 10 days before the day of infection (Figure 2A). The percentage of infected erythrocytes gradually increased with or without gum arabic treatment. However, it was slightly lower in gum arabic-treated animals than in animals without gum arabic treatment, an effect reaching statistical significance throughout 10-21 days of infection (Figure 2B).

Infection with *P. berghei *significantly decreased the erythrocyte number per μl, haematocrit (packed cell volume) and blood haemoglobin concentration (Table 1), effects all significantly blunted by treatment with GA (Table 1).

**Figure 3:**
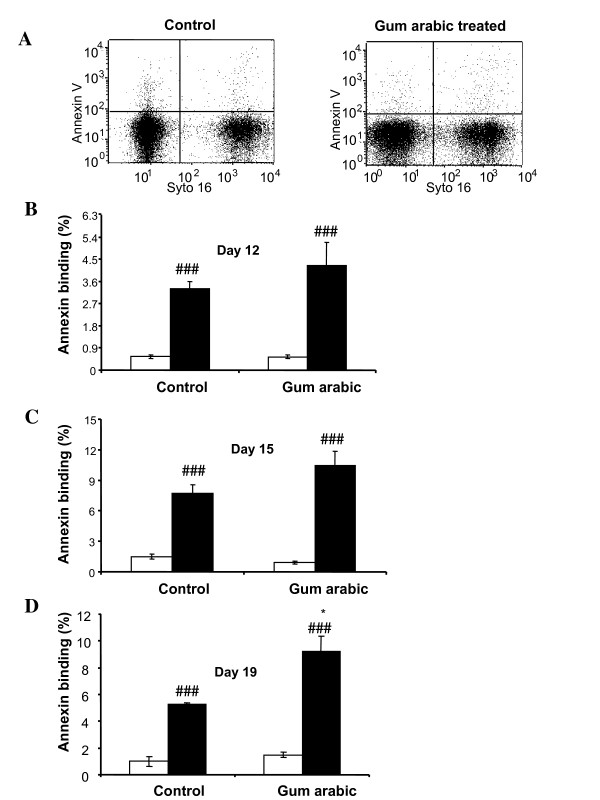
**Effect of gum arabic treatment on phosphatidylserine exposure of infected and noninfected erythrocytes in *Plasmodium berghei*-infected mice**. **A**: Original dot blot of annexin V-binding versus Syto 16 fluorescence in untreated animals (left panel) and animals treated with 10% gum arabic in drinking water (right panel) 19 days after infection with *P. berghei*. **B-D**. Arithmetic means ± SEM of the percentage of non-infected (white bars) and infected (black bars) annexin V-binding erythrocytes drawn from mice without (control, left bars) and with (GA, right bars) gum arabic treatment (10% gum arabic in drinking water) 12 (**B**), 15 (**C**) and 19 (**D**) days after infection with *P. berghei*. * (p ≤ 0.05) indicates significant difference from absence of gum arabic, ^### ^(p < 0.001) indicate significant difference from noninfected erythrocytes.

The infection was paralleled by triggering of cell membrane scrambling, as evidenced from annexin V binding (Figure 3). Irrespective of GA treatment, the percentage of annexin V binding cells was significantly higher in infected than in non-infected erythrocytes. The percentage of infected erythrocytes binding annexin V was higher in the GA-treated animals, an effect reaching statistical significance at day 19 of post-infection.

**Figure 4:**
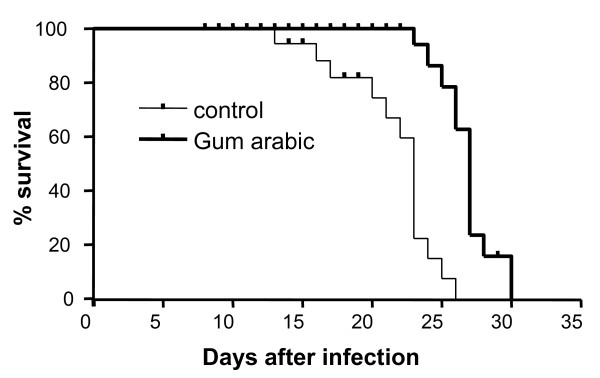
**Effect of gum arabic treatment on survival of *Plasmodium berghei *infected mice**. Survival of mice without treatment (light line) or with 10% gum arabic in drinking water (dark line) as a function of days after infection with *Plasmodium berghei*. Gum arabic treatment significantly (p < 0.0001, Kaplan-Meier survival function test) enhances the survival of infected mice.

The treatment with GA further influenced the survival of *P. berghei*-infected mice. As shown in Figure 4, all untreated animals died within 26 days after the infection. In contrast, as many as 70% of the GA-treated animals survived the infection for more than 26 days.

## Discussion

The present study reveals a completely novel effect of gum arabic, i.e. an influence on the course of malaria. Treatment with GA delayed a lethal course of malaria following infection of mice with *P. berghei*. As shown earlier, the infection of mice with *P. berghei *is followed by an invariably lethal course of malaria [62]. Treatment with GA did not prevent a lethal course of malaria but extended the survival of the infected animals. Accordingly, when all untreated animals had died, still more than half of the GA treated animals were alive.

The present observations do not allow safe conclusions as to the mechanisms underlying the moderate beneficial effect of GA treatment. Gum arabic treatment delayed the development of parasitaemia and blunted the decrease of blood erythrocyte number and haemoglobin concentration and thus significantly counteracted the development of anemia.

In theory, GA could affect parasitaemia and host survival by increasing the erythrocyte content of foetal haemoglobin, which is known to delay the intraerythrocytic growth of the parasite [17,18]. The effect would be apparent particularly following pretreatment of the mice with GA. While butyrate requires excessive concentrations to be effective *in vitro*, much lower concentrations could modify the formation of foetal hamoglobin [13-15] and thus susceptibility to malaria [16-18].

Foetal hemoglobin (HbF) has a higher O_2 _affinity than adult haemoglobin [64] and influences erythrocyte K^+ ^transport and O_2 _dependence of erythrocyte glycolysis [65]. Increased HbO_2 _affinity may result in enhanced lactate formation with subsequent decrease of HCO_3_^- ^and thus increased CO_2_/HCO_3_^- ^ratio. CO_2 _fosters SOD1 peroxidation, promoting the release of pro-inflammatory cytokines from activated macrophages leading to metabolic syndrome [66]. Those events may affect erythrocyte survival in parasitized erythrocytes.

Gum arabic, butyrate and/or foetal haemoglobin may affect parasitaemia and host survival by accelerating the suicidal death of infected erythrocytes [67]. Phosphatidylserine-exposing erythrocytes are phagocytosed [41,42] and thus rapidly cleared from circulating blood [43]. Eryptosis is triggered by a wide variety of substances [68-74]. Several of those substances have been shown to decrease parasitaemia and to extend the survival of infected mice [52,75-78]. Moreover, eryptosis is enhanced in several clinical conditions, such as iron deficiency [43], sickle-cell anaemia [79,80], beta-thalassaemia [22], glucose-6-phosphate dehydrogenase (G6PD)-deficiency [22], phosphate depletion [81], Haemolytic uremic syndrome [82], sepsis [83], malaria [45] and Wilson's disease [84]. Some of those diseases have similarly been shown to favourably influence the course of malaria, i.e. sickle-cell trait, beta-thalassaemia-trait, homozygous Hb-C and G6PD-deficiency [22,46-50] as well as iron deficiency [21]. However, most of those diseases and substances exert a profound effect on parasitaemia.

In conclusion, in mice, gum arabic provides extended survival following the invariably lethal infection with *P. berghei*. Gum arabic is particularly effective in preventing an early death from this devastating disease.

## Competing interests

The authors declare that they have no competing interests.

## Authors' contributions

AB performed in vitro experiments, DB performed in vivo experiments, SMQ analysed eryptosis, MF evaluated the data and made the statistical analysis, DK supervised the in vivo experiments, ON initiated the study, AS initiated the gum arabic research and participated in the design, FL wrote the manuscript. All authors read and approved the final manuscript.
